# A rapidly evolving single copy histone H1 variant is associated with male fertility in a parasitoid wasp

**DOI:** 10.3389/fcell.2023.1166517

**Published:** 2023-05-31

**Authors:** Bo Yuan, Yi Yang, Zhichao Yan, Chun He, Yu H. Sun, Fei Wang, Beibei Wang, Jiamin Shi, Shan Xiao, Fang Wang, Qi Fang, Fei Li, Xinhai Ye, Gongyin Ye

**Affiliations:** ^1^ State Key Laboratory of Rice Biology and Breeding and Ministry of Agricultural and Rural Affairs Key Laboratory of Molecular Biology of Crop Pathogens and Insects, Institute of Insect Sciences, Zhejiang University, Hangzhou, China; ^2^ Department of Biology, University of Rochester, Rochester, NY, United States; ^3^ Shanghai Institute for Advanced Study, Zhejiang University, Shanghai, China; ^4^ College of Computer Science and Technology, Zhejiang University, Hangzhou, China

**Keywords:** parasitoid wasp, *Pteromalus puparum*, histone H1 variant, chromatin, spermatogenesis

## Abstract

The linker histone H1 binds to the nucleosome core particle at the site where DNA enters and exits, and facilitates folding of the nucleosomes into a higher-order chromatin structure in eukaryotes. Additionally, some variant H1s promote specialized chromatin functions in cellular processes. Germline-specific H1 variants have been reported in some model species with diverse roles in chromatin structure changes during gametogenesis. In insects, the current understanding of germline-specific H1 variants comes mainly from the studies in *Drosophila melanogaster*, and the information on this set of genes in other non-model insects remains largely unknown. Here, we identify two H1 variants (*PpH1V1* and *PpH1V2*) that are specifically predominantly expressed in the testis of the parasitoid wasp *Pteromalus puparum*. Evolutionary analyses suggest that these H1 variant genes evolve rapidly, and are generally maintained as a single copy in Hymenoptera. Disruption of *PpH1V1* function in the late larval stage male by RNA interference experiments has no phenotype on spermatogenesis in the pupal testis, but results in abnormal chromatin structure and low sperm fertility in the adult seminal vesicle. In addition, *PpH1V2* knockdown has no detectable effect on spermatogenesis or male fertility. Collectively, our discovery indicates distinct functions of male germline-enriched H1 variants between parasitoid wasp *Pteromalus* and *Drosophila*, providing new insights into the role of insect H1 variants in gametogenesis. This study also highlights the functional complexity of germline-specific H1s in animals.

## Introduction

Eukaryotic genomes are packed in the form of chromatin within the cell nucleus, and the linker histone H1 protein is one of the main components of eukaryotic chromatin, which binds to adjacent nucleosomes and linker-DNA, facilitating the forming of the higher-order chromatin structure known as the chromatin fiber (Fan et al., 2005; Harshman et al., 2013; Fyodorov et al., 2018). The H1 gene family has evolved faster than other core histone families (H2A, H2B, H3, and H4), which has contributed to the emergence of many H1 variants with redundant or specific functions (Izzo et al., 2008; Ponte et al., 2017). Among the diverse H1 variants, a group exhibits a highly-germline-enriched expression pattern, and plays key roles in changing the chromatin structure during gametogenesis and early embryogenesis (Pérez-Montero et al., 2016). In mammalians, a total of eleven H1 variants are identified, seven of which are somatic subtypes (H1.0, H1.1 to H1.5, and H1x), and the additional four are germline-specific subtypes: H1t, H1T2, HILS1 in the testis (Kumaroo and Irvin, 1980; Seyedin and Kistler, 1980; Kremer and Kistler, 1991; Drabent et al., 1993; Yan et al., 2003; Martianov et al., 2005; Tanaka et al., 2005), and H1oo in the oocyte (Tanaka et al., 2001). In general, germline-specific H1s are strictly regulated during gametogenesis and they may function in different stages. For example, H1t is the first variant to be expressed in meiotic spermatocytes and compacts chromatin to a lesser extent and binds DNA with lower affinity, which may facilitate meiotic recombination (De Lucia et al., 1994; Khadake and Rao, 1995; Talaszt et al., 1999). Later, H1T2 and HILS1 express and gradually replace H1t, which contributes to the compaction of sperm chromatin during spermatid stages (Iguchi et al., 2003; Yan et al., 2003; Martianov et al., 2005; Tanaka et al., 2005). Other germline-specific H1s were also identified in some model species in other taxa, such as zebrafish (H1M) (Müller et al., 2002), clawed frog (H1fx and B4/H1M) (Smith et al., 1988; Shechter et al., 2009), sea urchin (SpH1 and Cs-H1) (Strickland et al., 1980; Brandt et al., 1997; Mandl et al., 1997), and nematode (H1.1/HIS-24) (Vanfleteren et al., 1988). These studies showed that, although these variants are largely restricted to the germline, they may have distinct expression pattern and different functions (Pérez-Montero et al., 2016), suggesting the high complexity of germline-specific H1s in animals.

Insects comprise extremely diverse species and are important models for understanding the functional diversification of genes, such as chemosensory-related gene families (Eyun et al., 2017; Robertson, 2019). To date, our ideas of insect germline-specific H1s are solely from studying the fruit fly *Drosophila melanogaster*. A germline-specific H1 variant dBigH1 has been reported (Pérez-Montero et al., 2013), which is expressed in both the female and male germlines, and early embryos. dBigH1 is abundant during early embryogenesis and regulates zygotic genome activation (Pérez-Montero et al., 2013). In male, dBigH1 is essential for germ stem cell (GSC) maintenance and spermatocyte differentiation (Carbonell et al., 2017). Another H1-like linker protein Mst77F is detected from late canoe stage spermatids to mature sperm overlapping with protamines. And *Mst77F* mutants show low fertility and morphologically abnormal spermatids (Kimura and Loppin, 2016). Although the studies on model organism *Drosophila* greatly advance our knowledge of germline-specific H1 variants in insects, the relevant information of this group of genes in other non-model insects, including their sequences, origins, evolutionary trajectories and their biological functions, is poorly understood.

In this study, we identify two male germline-enriched histone H1 variants (*PpH1V1* and *PpH1V2*) in the model parasitoid wasp, *Pteromalus puparum* (from Hymenoptera). Our evolutionary analyses show that these two H1 variant genes generally keep as a single copy in all hymenopterans we examined, although they evolve rapidly. We knockdown *PpH1V1* and *PpH1V2* using RNA interference (RNAi), and demonstrate that *PpH1V1* may play a critical role in the chromatin organization of sperm in the seminal vesicle, and is associated with male fertility. However, *PpH1V2* knockdown has no detectable effect on spermatogenesis or male fertility. This study provides new insights into the role of H1 variants in gametogenesis, and contributes to expanding our understanding of insect H1 variants in non-model species.

## Results

### Two rapidly evolving histone H1 variants in *P. puparum*

**FIGURE 1:**
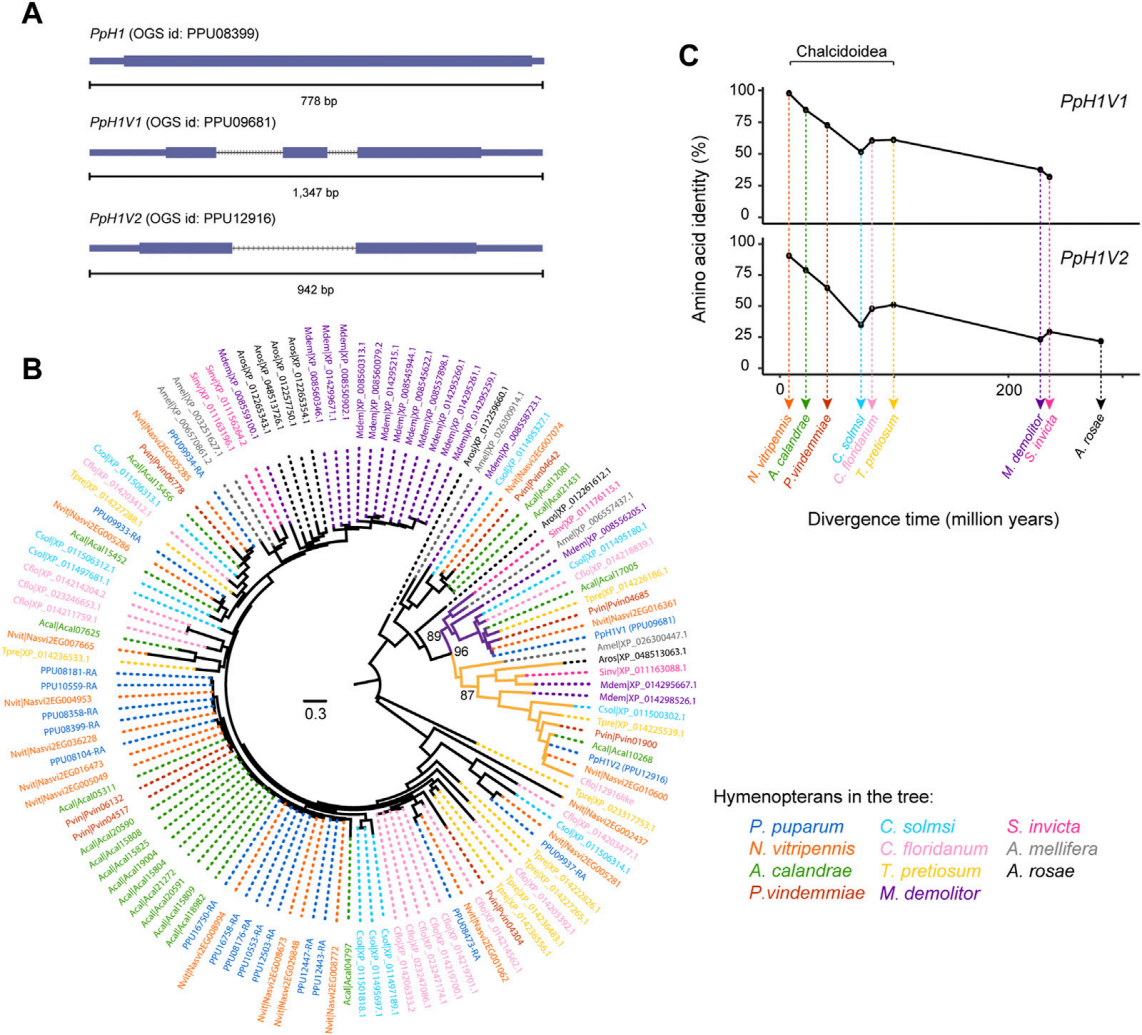
Two rapidly evolving histone H1 variants in *P. pupraum* genome. **(A)** Comparison of the gene structures of a generic histone H1 gene and two histone H1 variant genes (*PpH1V1* and *PpH1V2*). The coding exons are shown as blocks connected by horizontal lines representing introns, and the 5′and 3′untranslated regions (UTRs) are shown as thinner blocks. **(B)** A maximum-likelihood protein phylogeny of the histone H1 sequences from 11 representative hymenopteran species. The clade containing PpH1V1 was highlighted in purple, and the clade with PpH1V2 was in yellow. Statistical support for the phylogeny was assessed by ultrafast bootstrap analysis using 1,000 replicates, and bootstrap values at selected nodes were indicated. **(C)** Pairwise amino acid identity analysis showing comparisons of PpH1V1 or PpH1V2 *versus* the hymenopteran ortholog of each variant. Percent identities were plotted against species divergence time. The species divergence times were obtained from previous studies (Peters et al., 2017; Ye et al., 2022).

The *P. puparum* genome sequencing project identified 18 histone H1 genes in the official gene set (OGS) by an automatic genome annotation pipeline which integrated the evidences from homology mapping, *de novo* prediction and gene expression (Ye et al., 2020). In this study, to obtain a full list of H1 genes in *P. puparum*, we performed a comprehensive homology-based search using the previously identified H1 sequences. We further determined the presence of the functional domain (Pfam id: PF00538, Linker_histone) and checked the complete open reading frame for each candidate. In total, we confirmed the accurate gene annotation of H1 genes in OGS, and did not find additional genes which were miss-annotated in our previous genome annotation. Among these 18 genes, two were further assigned as putative H1 variants, according to the HistoneDB 2.0 online service (Draizen et al., 2016). We thus named them as *P. puparum* H1 variant 1 (*PpH1V1*, OGS id: PPU09681) and *P. puparum* H1 variant 2 (*PpH1V2*, OGS id: PPU12916). Gene structure analysis showed that both putative H1 variants contain introns (Figure 1A), which is distinctly different from generic histone H1 (such as *PpH1*, OGS id: PPU08399) that lack introns (Hentschel and Birnstiel, 1981). In addition, previous studies have shown that the insertion of an intron into a histone gene could alter the histone 3′end formation and thus result in the formation of polyadenylated mRNA (Pandey et al., 1990). As expected, we used rapid amplification of cDNA ends (RACE) to amplify the full-length mRNAs and confirmed the existence of poly(A) tails in the mRNAs of these H1 variants (Supplementary Table S1). Sequence analysis of the *PpH1V1* full length mRNA (1,093 base pair, bp) revealed a 651 bp open reading frame encoding a 216 amino acid protein of predicted molecular mass 24.24 kDa and the *PpH1V2* full length mRNA (698 bp) revealed a 444 bp open reading frame encoding a 147 amino acid protein of predicted molecular mass 16.04 kDa (Supplementary Figure S1).

To understand the evolutionary history of H1 genes in Hymenoptera, we identified the H1 genes in other ten hymenopteran species with high-quality genomes, representing parasitoid wasp, ant, bee, and sawfly lineages. Then, we performed maximum likelihood phylogenetic analysis with protein sequences. Our analysis revealed that *PpH1V1* and *PpH1V2* belonged to two distinct clades, and both clades were overall broadly distributed among hymenopterans (Figure 1B). The genes in these clades generally kept as a single copy in all hymenopterans we examined, except *Microplitis demolitor*. In addition, the two H1 variant clades were further clustered together with high bootstrap support (96%), suggestive of the evolutionary origin of one of the H1 variants by an ancient gene duplication event. Ortholog inference based on reciprocal mapping, phylogenetic tree and synteny could find an unambiguous ortholog of *PpH1V2* in all hymenopterans we examined; however, we failed to infer the unambiguous ortholog of *PpH1V1* in *Athalia rosae* (early branch of Hymenoptera), because the candidate gene (XP_012261612.1) was not clustered with other orthologs of *PpH1V1*, and there was no synteny signal. We next investigated the protein divergence rates of these H1 variants in Hymenoptera spanning 281 million years of evolution. To this end, we measured the pairwise identity of each H1 variant protein to its ortholog of *P. puparum* and plotted it with the species divergence time. We showed that the pairwise amino acid identity decreased rapidly over the divergence time (from 97% to 32% for *PpH1V1*, and from 92% to 22% for *PpH1V2*), reflecting the rapid evolution of these H1 variant proteins (Figure 1C).

### *PpH1V1* and *PpH1V2* are expressed predominantly in the testis

**FIGURE 2:**
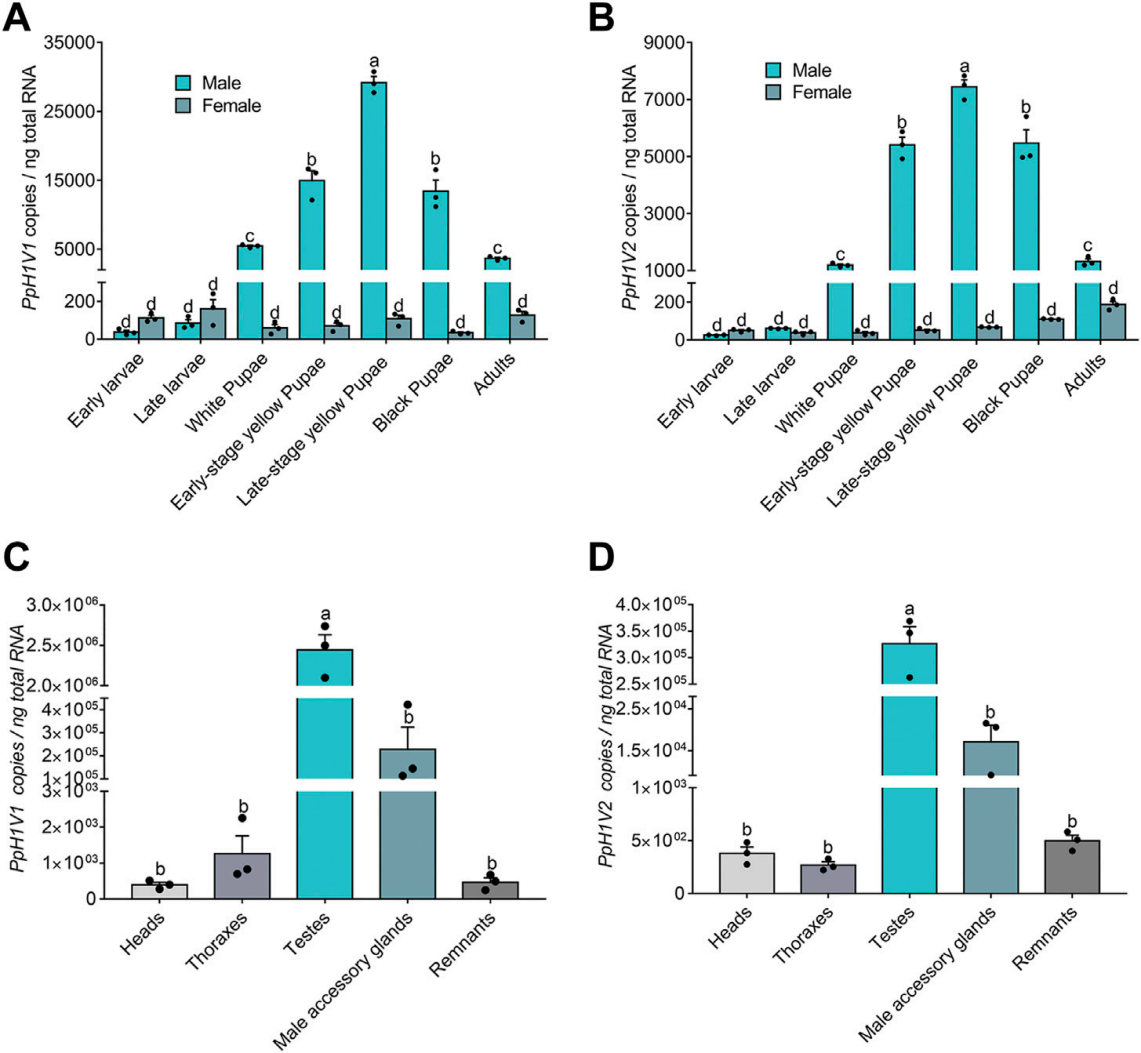
Expression patterns of *PpH1V1* and *PpH1V2* mRNA in different developmental stages and different tissues. **(A–B)** Expression levels of *PpH1V1* gene **(A)** and *PpH1V2* gene **(B)** in different developmental stages (from the larval stage to the adult stage, in both sexes). **(C–D)** Expression levels of *PpH1V1* gene **(C)** and *PpH1V2* gene **(D)** in different tissues of the late-stage yellow pupal males. For each measurement, three biological replicates were done. The qRT-PCR data were presented as mean ± standard error. Differences between groups were analyzed by one-way ANOVA with Tukey’s multiple comparisons test, and bars annotated with the same letters are not significantly different (*p* > 0.05).

We next sought to investigate the expression of the two H1 variants in *P. puparum*. First, we performed quantitative real-time PCR (qRT-PCR) for each variant on different developmental stages (totally seven time points from early larva to adult) of both sexes. The results showed that these two H1 variants were lowly expressed (34–161 and 38–110 copies per ng total RNA, respectively) in females, and there were no significant expression differences during the female development. By contrast, the expressions of these H1 variants were much higher (38–29,198 and 26–7,450 copies per ng total RNA, respectively) in males (Figures 2A, B). Importantly, we found that, from the late larval stage to the late yellow pupal stage, these H1 variants steadily increased their expressions with statistically significant differences (Figures 2A, B, *p* < 0.0001, Tukey’s multiple comparisons test), resulting in the highest expression level at the late yellow pupal stage. Then, the mRNA levels of these H1 variants decreased gradually from the late yellow pupal stage to the adult stage. These male-biased, and developmental-stage-biased gene expression patterns prompted us to test whether these H1 variants are highly specific to the germline and may be related to spermatogenesis, because the expression patterns are highly consistent with the spermatogenesis process in parasitoid wasps (Ferree et al., 2019). Next, we measured the expressions of these H1 variants on a representative set of tissues (head, thorax, testis, male accessory gland and body remnant) from the late-stage yellow pupal males. As expected, although the expressions were detectable in other tissues, we observed the significantly higher expressions of these H1 variants in the testis (Figures 2C, D, *p* < 0.0001, Tukey’s multiple comparisons test). To investigate the immunolocalization of PpH1V1 and PpH1V2 in the germ cells, we raised polyclonal antibodies against them, separately. We observed that spermatids with elongating nuclei were specifically stained by both antibodies (Supplementary Figures S2A, B). Taken together, our results indicate that both H1 variants showed the male-biased, and testis-specific high expression patterns, implying their potential roles in male fertility.

### Knockdown of *PpH1V1* in male induces significantly lower sex ratio in offspring

**FIGURE 3:**
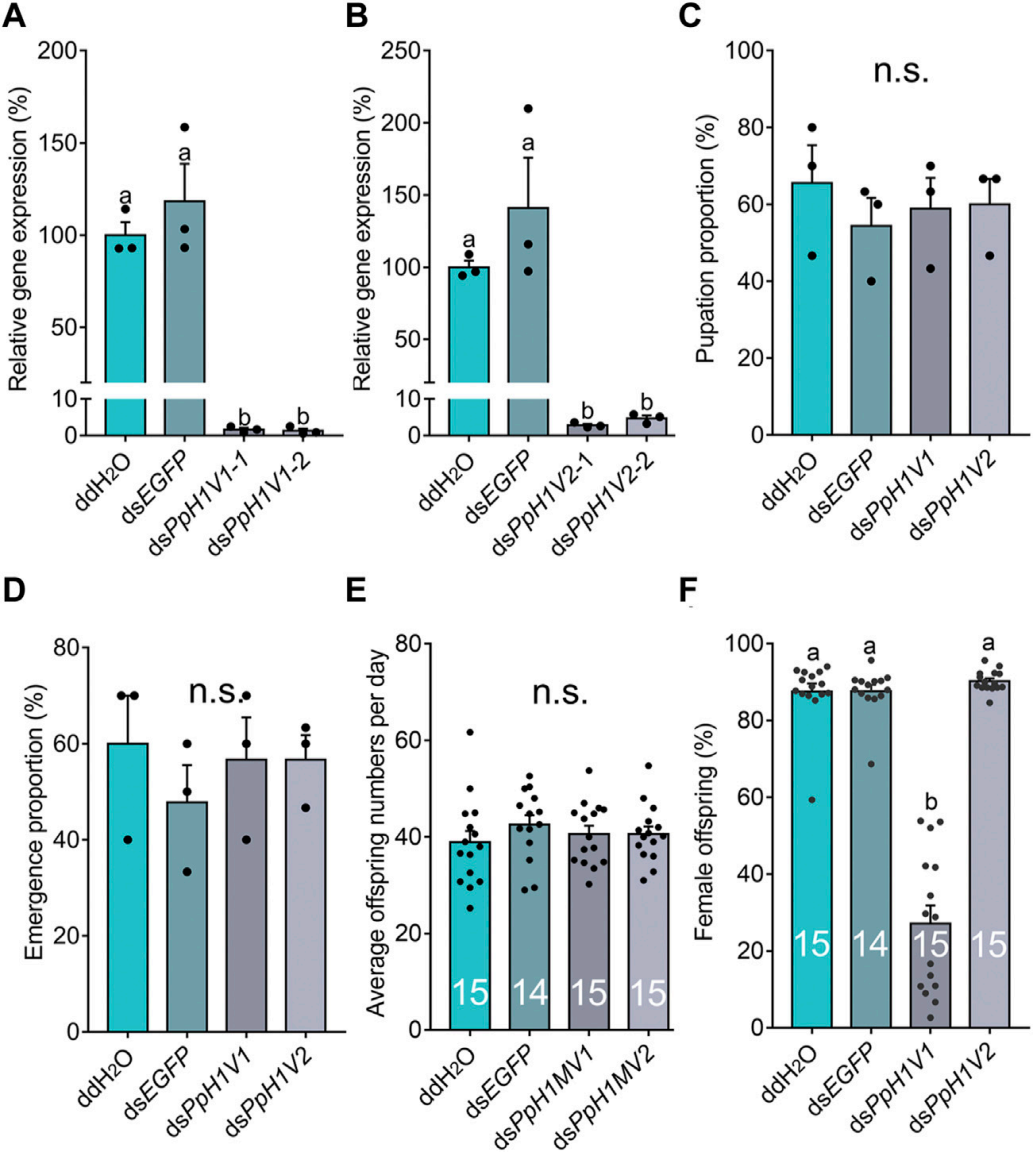
Knockdown of *PpH1V1* in males results in significantly lower sex ratio in offspring. **(A–B)** Expressions of *PpH1V1* **(A)** and *PpH1V2* **(B)** were successfully knocked down in pupal males, three biological replicates. **(C–D)** Knockdown of *PpH1V1* or *PpH1V2* in male caused no effect on pupation proportion **(C)** or emergence proportion **(D)**, sample size = 10, three biological replicates. **(E)** Knockdown of *PpH1V1* or *PpH1V2* in male resulted in no effect on average offspring number after mating with one female, sample size = 14 or 15. **(F)** Knockdown of *PpH1V1* in male leaded to the significantly decrease of the sex ratio (female/male) of offspring, sample size = 14 or 15. All values were presented as mean ± standard error. Differences between groups were analyzed by one-way ANOVA with Tukey’s multiple comparisons test. Bars annotated with the same letters are not significantly different (*p* > 0.05); n.s. not significantly different.

**FIGURE 4:**
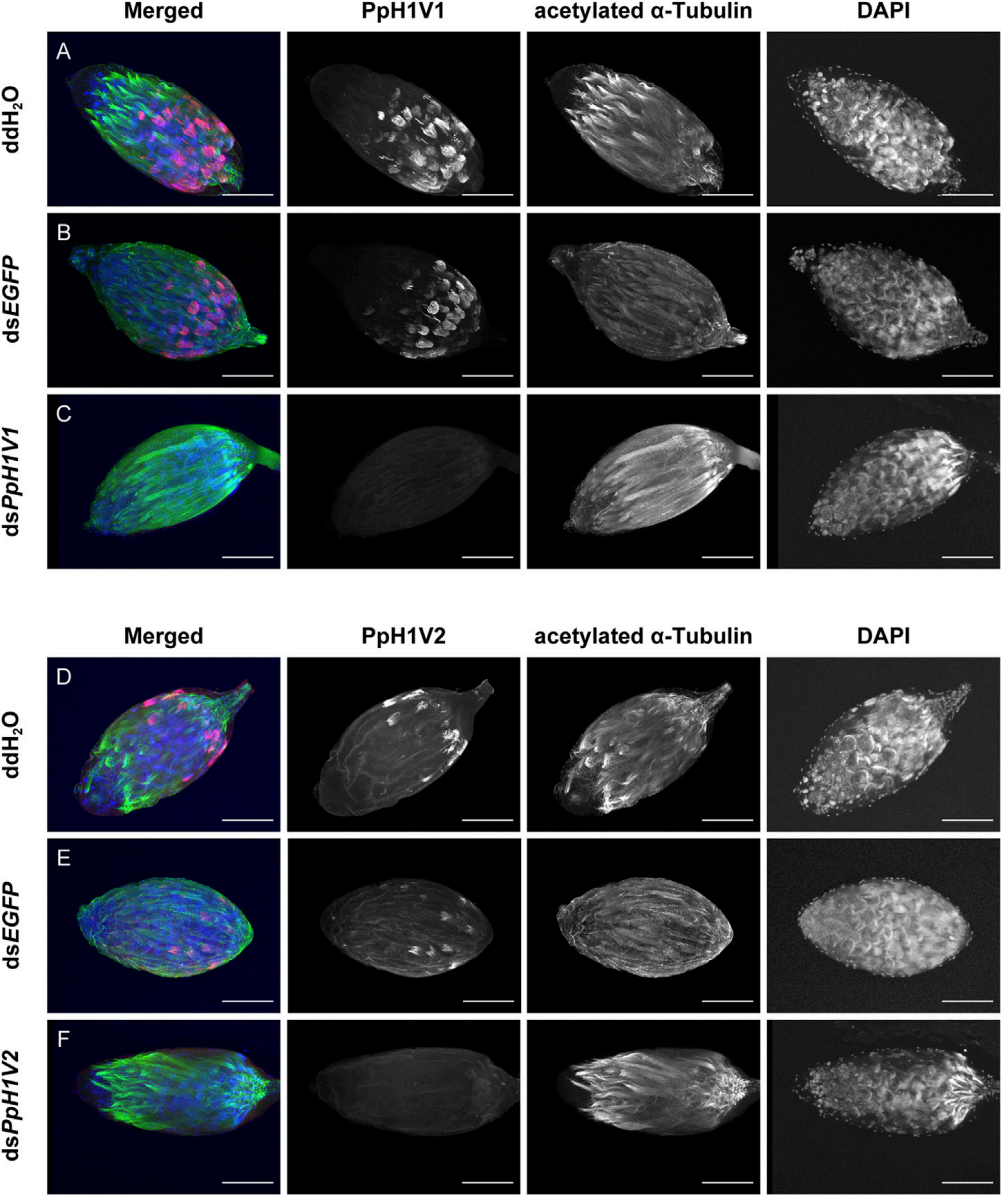
RNA interference (RNAi) silencing effects on PpH1V1 and PpH1V2 at the protein levels. Immunofluorescence staining showing the testes of control and knockdown RNAi yellow-black pupal males. PpH1V1 **(A–C)** or PpH1V2 **(D–F)** was detected using a rabbit anti-PpH1V1 or a rabbit anti-PpH1V2 polyclonal antibody and goat anti-rabbit IgG (H + L) with Alexa Fluor™ 488 secondary antibody (shown in red). Flagellum was stained with acetylated α Tubulin Alexa Fluor^®^ 647 antibody (shown in green). Nuclei was stained with DAPI (shown in blue). Images are shown in greyscale for single channels. Three biological replicates were performed for each measurement. Scale bars correspond to 100 μm.

Next, we knocked down the two H1 variants by injecting dsRNA into the male larvae. To reduce the likelihood of false positives due to sequence-specific off-target effects, we used two independent, non-overlapping dsRNAs against *PpH1V1* and *PpH1V2*. At the late yellow pupal stage (5 days post injection), the transcript abundance of ds*PpH1V1* and ds*PpH1V2* treated wasps were significantly decreased compared to the negative controls (over 95% reductions), which were treated with ddH_2_O and ds*EGFP* (*EGFP*: enhanced green fluorescent protein gene), respectively (Figures 3A, B, *p* < 0.0001 and *p* < 0.001, Tukey’s multiple comparisons test). RNAi effects were also evaluated at the protein levels, and the immunofluorescence staining analysis revealed strong reductions of PpH1V1 (Figures 4A–C) and PpH1V2 (Figures 4D–F) protein contents in the dsRNA treated wasps, respectively. Then, the dsRNA and negative control treated wasps were kept for phenotype observation until emergence, and we did not observe significant differences in either pupation proportion or emergence proportion between the dsRNA treated groups and the negative controls (Figures 3C, D, *p* = 0.8094 and *p* = 0.7464, Tukey’s multiple comparisons test). To test whether these two H1 variants play important roles in male fertility, we paired the dsRNA treated or negative control male adults with virgin female adults, and allowed them to mate for 48 h, then put females individually in glass tubes with one newly-pupated host. During the following 5 days, each host encountered by the female wasp was individually transferred into a new glass tube every day, and the fresh pupa and food were provided and replaced. According to the special haplodiploidy sex-determination system of parasitoid wasps, males develop from unfertilized eggs and are haploid, and females develop from fertilized eggs and are diploid (Cockburn, 1993; Heimpel and De Boer, 2008). Therefore, theoretically, mating with the male wasps that are infertile would result in fertilization failure, and thus induce more male offspring and lower offspring sex ratio (number of female offspring/total number of offspring). Indeed, our results revealed that the dsRNA treatments had no influence on the offspring number (Figure 3E), but knockdown of *PpH1V1* in males induced a significantly lower offspring sex ratio (27%) than those in the control groups (87% for ddH_2_O and 87% for ds*EGFP*) (Figure 3F, *p* < 0.0001, Tukey’s multiple comparisons test), suggesting the crucial role of *PpH1V1* in male fertility. However, we did not find any significant effects on offspring sex ratio in ds*PpH1V2* treatment compared to the controls (Figure 3F).

### Knockdown of *PpH1V1* does not interrupt spermatogenesis in testis

**FIGURE 5:**
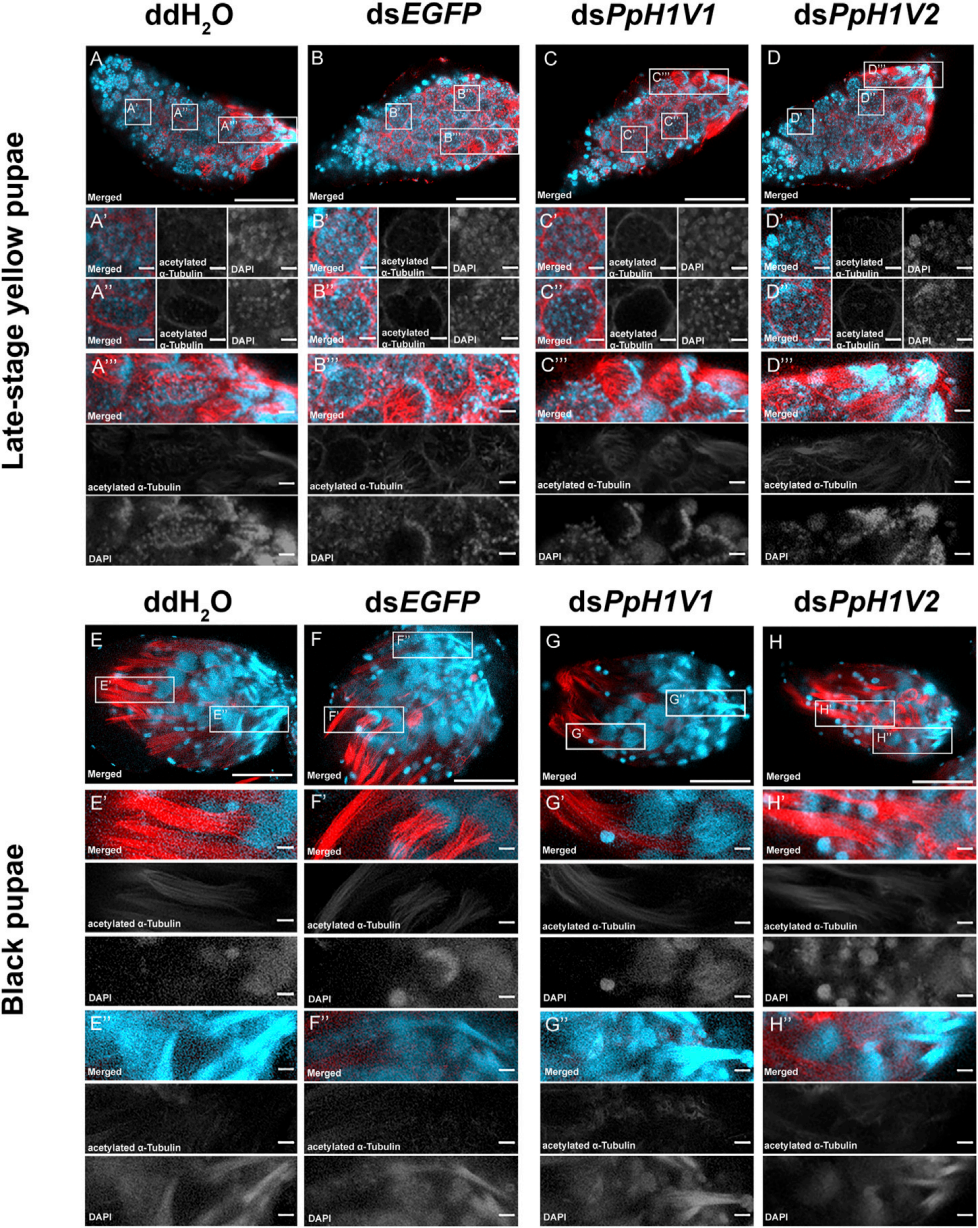
*PpH1V1* knockdown does not affect the spermatogenesis in the testis. **(A–D)** Immunofluorescence staining showing the testes of late-stage yellow pupal males under four different treatments. **(A′–D′)** Small cyst of germ cells at the apical end of the testis. **(A′′–D′′)** Germ cells in a more posterior-located cyst have entered into spermiogenesis, as indicated by their condensed nuclei. **(A‴–D‴)** Bundles of spermatids with elongating tails. **(E–H)** Immunofluorescence staining showing the testes of black pupal males under four different treatments. **(E′–H′)** Bundles of spermatids with elongating nucleus. **(E′′–H′′)** Bundles of sperm with fully elongated sperm nucleus. Flagellum was stained with acetylated α Tubulin Alexa Fluor^®^ 488 antibody (showed in red). Nucleus was stained with DAPI (shown in light blue). Images are shown in greyscale for single channels. Three biological replicates were performed for each measurement. Scale bars correspond to 100 μm in **(A-H)** and 10 μm in all others.

We next tested whether *PpH1V1* was involved in the spermatogenesis of *P. puparum* males. For this purpose, we first examined the spermatogenesis process in the testes of RNAi-treated late-stage yellow pupal males. At this developmental stage, we observed several cells at distinct status, for example, germ cells at interphase (Figure 5A’), germ cells with condensed nuclei (Figure 5A’’) which entry into spermiogenesis (Supplementary Figure S3), and germ cells reorganized into a hemispherical shape (cup-like) (Figure 5A’’’). Compared to ddH_2_O and ds*EGFP* treatments (Figures 5A, B), we did not find obvious disorders of spermatogenesis in the ds*PpH1V1*-treated wasps, nor in the ds*PpH1V2*-treated groups (Figures 5C, D). The results from transmission electron microscopy (TEM) indicated that no phenotype during early spermatogenesis process in the testes of RNAi-treated wasps (Supplementary Figure S4A). Moreover, we did not find significant expression changes of a conserved spermatogenesis-related marker gene *Vasa* between RNAi-treated wasps and controls (Supplementary Figure S4B). In the older testes (black pupae), bundles of elongating spermatid nuclei or fully elongated sperm nuclei were detected in controls, ds*PpH1V1*- and ds*PpH1V2*-treated wasps (Figures 5E–H), suggesting that knockdown of these two H1 variants does not affect the spermiogenesis in testis.

### *PpH1V1* may be involved in sperm chromatin organization

**FIGURE 6:**
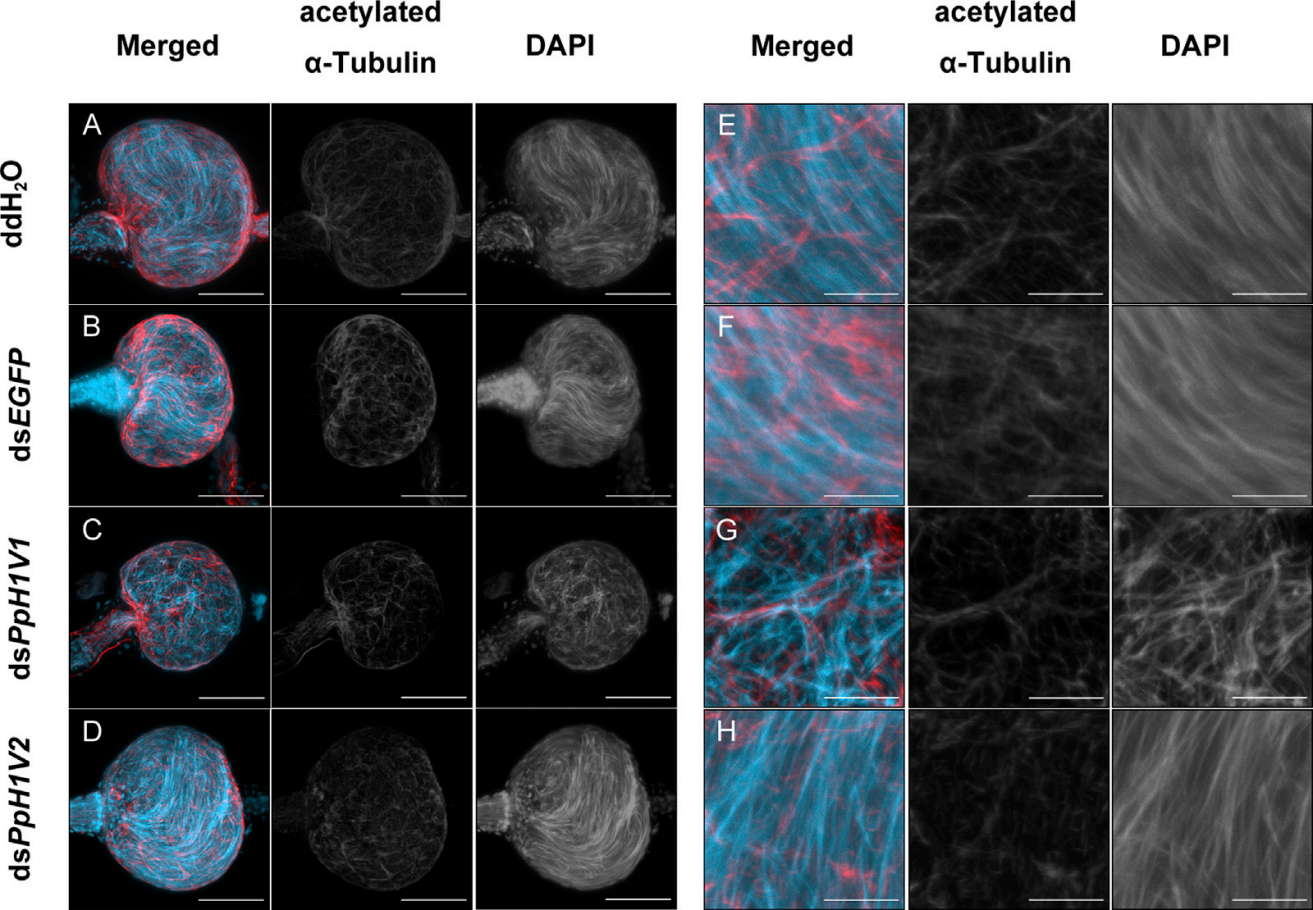
Knockdown of *PpH1V1* resulted in obvious abnormal sperm nuclei in the seminal vesicles of adult males. **(A–D)** Immunofluorescence staining showing the seminal vesicles of adult males under four different treatments. **(E–H)** The local enlarged images of **(A–D)**. Flagellum was stained with acetylated α Tubulin Alexa Fluor^®^ 488 antibody (showed in red). Nucleus was stained with DAPI (shown in light blue). Images are shown in greyscale for single channels. Three biological replicates were performed for each measurement. Scale bars correspond to 50 μm in **(A–D)** and 10 μm in **(E–H)**.

The above results can not explain why *PpH1V1* knockdown could influence male fertility and result in the change of sex ratio of offspring. We then asked whether *PpH1V1* was required for maintaining the chromatin organization of sperm in the seminal vesicle, a downstream organ of testis for sperm storage. In contrast to the well-organized needle-shaped nuclei of sperm in the seminal vesicles of adult wasps from the control treatments (Figures 6A, B, E, F), the nuclei of sperm in the seminal vesicles of ds*PpH1V1-*treated wasps were in disarray (Figures 6C, G), indicating potential disorder in the sperm chromatin. In addition, we investigated the nuclear morphology of sperm in seminal vesicles of RNAi-treated wasps and controls. We observed that the sperm nuclei of the control treatments were needle-like, while the sperm nuclei of the ds*PpH1V1*-treated wasps were clearly deformed and irregular in shape, with short but bent or crumpled nuclei (Supplementary Figure S5). In contrast, we did not find such abnormal chromatin status in the sperm from ds*PpH1V2*-treated wasps (Figures 6D, H; Supplementary Figure S5). Using transmission electron microscopy (TEM), we did not observe a significant reduction in sperm density in the seminal vesicles of ds*PpH1V1*-treated wasps compared to the control treatments (Supplementary Figure S6).

**FIGURE 7:**
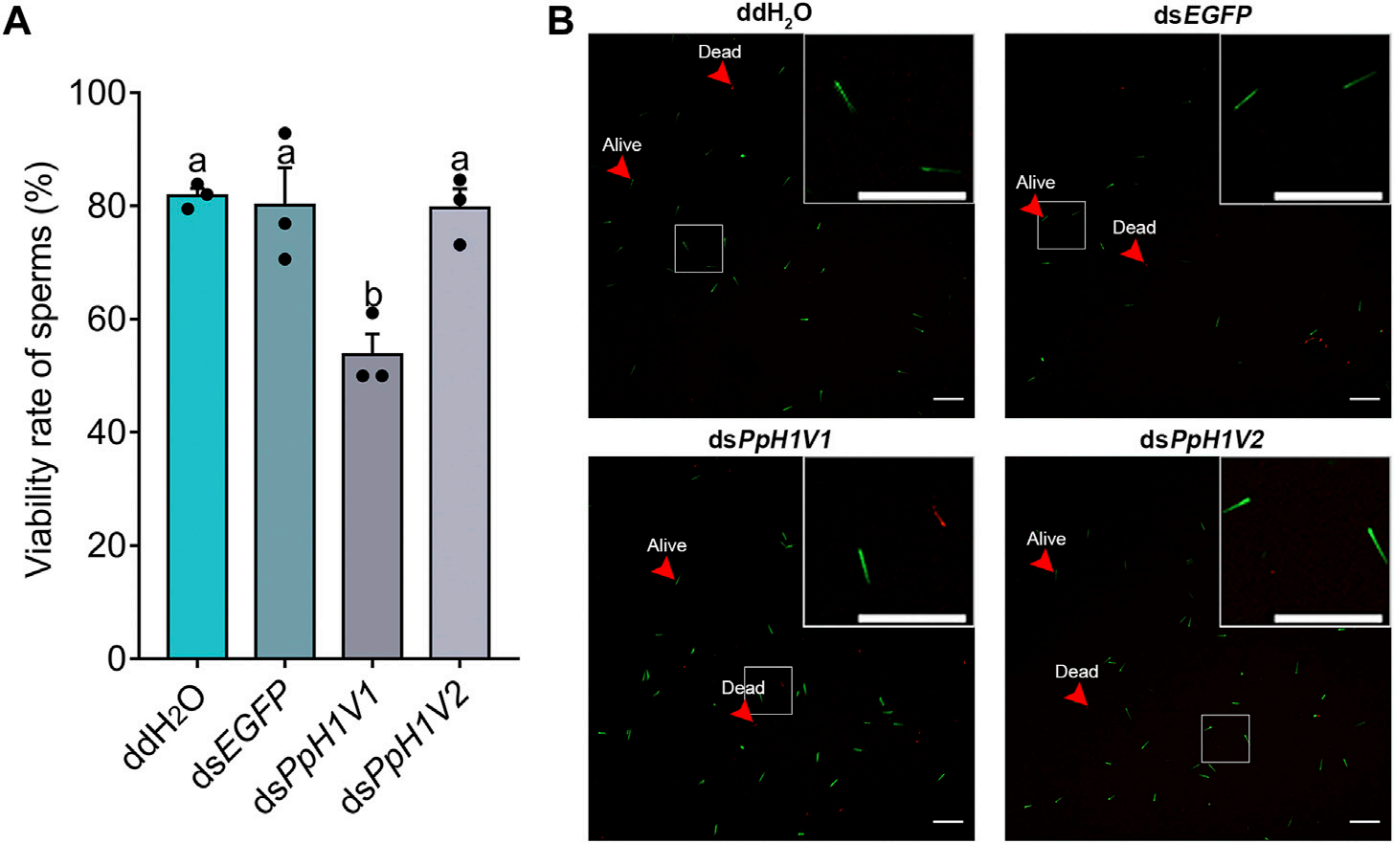
Sperm viability was significantly reduced in *PpH1V1* knockdown males. **(A)** Viability rate of sperm in the seminal vesicles of adult males. Three biological replicates were performed for each measurement. All values were presented as mean ± standard error. Differences between groups were analyzed by one-way ANOVA with Tukey’s multiple comparisons test. Bars annotated with the same letters are not significantly different (*p* > 0.05). **(B)** Microscopic image of sperm. Live and dead sperm were shown in green and red, respectively. Red arrows indicate examples of sperm that were alive or dead. Scale bars correspond to 100 μm.

We also performed a sperm viability assay to test whether *PpH1V1* knockdown induced more dead sperm than controls. Indeed, the results revealed that knockdown of *PpH1V1* in males induced significantly lower sperm viability (54%) than those in control groups (82% for ddH_2_O and 80% for ds*EGFP*) (Figures 7A, B, *p* < 0.0001, Tukey’s multiple comparisons test). And *PpH1V2* knockdown did not affect the sperm viability compared to controls. Altogether, our results suggest that *PpH1V1* is likely to be involved in the chromatin organization of sperm, and thus plays a critical role in male fertility in *P. puparum*.

## Discussion

Germline-enriched histone H1 variants have been reported in many metazoan species, and studies on the model species of some key taxa have revealed that the germline-enriched H1 variants play crucial roles in gametogenesis (Pérez-Montero et al., 2016). In insects, one of the most diverse groups of animals on Earth, the knowledge of germline-enriched H1 variants was limited to *Drosophila* species (Carbonell et al., 2017; Climent-Cantó et al., 2021), and very little is known about these variants in other insects. In this study, we reported two testis-specific highly expressed H1 variant genes (*PpH1V1* and *PpH1V2*) in the parasitoid wasp *P. puparum*, and functional studies suggested that *PpH1V1* is associated with male fertility in the wasp.

In *Drosophila*, a germline-enriched H1 variant (dBigH1) and three types of sperm nuclear basic proteins have been so far identified (Mst35Ba/b, Mst77F and Prtl99C), with different expression patterns and roles in spermatogenesis (Tirmarche et al., 2014; Eren-Ghiani et al., 2015; Kimura and Loppin, 2016; Carbonell et al., 2017). In particular, the expression of dBigH1 variant is largely restricted to spermatocytes, and it interplays with bag-of-marbles (Bam) to form a repressor loop that is essential for male germ stem cell differentiation (Carbonell et al., 2017). The loss of dBigH1 in germ cells of testes prevents spermatogonia from proliferation to differentiation, resulting in the accumulation of mitotic spermatogonia that fails to differentiate into spermatocytes and mature sperm (Carbonell et al., 2017). While Mst77F, which shows weak similarity to HILS1, expresses in elongating spermatids and is involve in shaping the nuclear of sperm (Jayaramaiah Raja and Renkawitz-Pohl, 2005; Rathke et al., 2010). Mst77F mutants show low fertility and morphologically abnormal spermatids (Jayaramaiah Raja and Renkawitz-Pohl, 2005; Kimura and Loppin, 2016). In this study, we showed that knockdown of *PpH1V1* resulted in abnormal chromatin patterns and low fertility sperm in the seminal vesicles, and this may be the cause of the observation of the significantly reduced male fertility. And we did not find obvious spermatogenesis interruption in testis. This result suggests that, like the *Mst77F* in *Drosophila*, *PpH1V1* may play a key role in the late process of spermatogenesis. However, although *PpH1V1* knockdown induced obviously disordered chromatin organization of sperm, mature sperm were still present in the seminal vesicles. This phenotype differs from that of Mst77F mutants in *Drosophila*, which had no mature sperm in the seminal vesicles (Kimura and Loppin, 2016). This finding suggests that, although PpH1V1 and Mst77F may involve in the late process of spermatogenesis, they have distinct functions. Additionally, PpH1V1 showed very weak protein sequence identity with Mst77F (24%), and Blastp against *Drosophila* proteins fails to find any hits, indicating no strong evidence of the orthologous relationship between two proteins. The functions of PpH1V2 variant remain still unclear, such as the knockdown revealed no detectable fertility defects or spermatogenesis interruption, although it carried a testis-specific expression pattern. A similar phenomenon was also found in mice, in which the loss of germline H1 do not reduce male fertility (Drabent et al., 2000; Lin et al., 2000; Fantz et al., 2001). Overall, our findings clearly revealed distinct functions of male germline-enriched H1 variants in parasitoid wasp and *Drosophila* (Kimura and Loppin, 2016; Carbonell et al., 2017), implying the highly diverged roles of this kind of H1 genes in insects.

As we mentioned above, male germline-enriched H1 variants are often detectable in a specific cell type or process during spermatogenesis. For example, in mammals, H1t generally expresses in meiotic spermatocytes and is also detected during spermatid differentiation, depending on the species (Seyedin and Kistler, 1980; Drabent et al., 1996; 1998; Steger et al., 1998), while HILS1 and H1T2 are restricted to spermatids (Iguchi et al., 2003; Yan et al., 2003; Martianov et al., 2005; Tanaka et al., 2005). In *Drosophila*, dBigH1 expression is tightly turned on in male germ stem cells and spermatocytes in the early stages of spermatogenesis (Carbonell et al., 2017), whereas Mst77F is detected together with protamines from late canoe stage spermatids to mature sperm (Rathke et al., 2010; Kimura and Loppin, 2016). Here our qPCR experiments revealed that, in the parasitoid wasp *P. puparum*, the two H1 variants were expressed predominantly in the testis. And their protein expression in the germ cells was demonstrated by immunolocalization. Moreover, we found that these proteins were detected in spermatids with elongating nuclei, but not in mature sperm with elongated nuclei, suggesting that these H1 variants are probably involved in restructuring of the chromatin of elongating spermatids and are subsequently replaced in mature sperm.

We found the two H1 variant genes generally kept as a single copy in all hymenopterans we examined. This discovery suggests *H1V1* gene could thus conservatively work as a key factor for male fertility in Hymenoptera. However, further evidence is needed to test this hypothesis by expanded sampling and functional studies. Protein sequence analyses showed rapid evolution of these H1 variants, consistent with the finding that H1 is the most divergent and heterogeneous group of histones (Zhang et al., 2012). Given this fast-evolving pattern, it is challenging to determine the origin and evolutionary trajectory of these two variant genes. Our gene scanning failed to detect putative orthologs of the two H1 variants outside Hymenoptera, thus we conclude that they either arose only in Hymenoptera, or there are not enough distinguishing sequence features for us to unambiguously identify their orthologs outside Hymenoptera. Phylogenomic analysis identified distinct H1V1 and H1V2 variant clades in Hymenoptera, and these two clades were clustered together, suggesting that one of the variants is likely raised by an ancient gene duplication.

In conclusion, we present two rapidly evolving male germline-enriched histone H1 variants in a parasitoid wasp, apparently representing the first view of germline-enriched H1 variants of non-model insects. We then demonstrate that one of the variants, *PpH1V1* is associated with male fertility. The roles of these variants in wasps are likely to be different from the H1 variants reported in *Drosophila*, suggesting the functional diversification of male germline-enriched H1 variants in insects. Thus, our study provides new insights into the role of H1 variants in gametogenesis, and paves the way for future functional and evolutionary studies of H1 variants in insects.

## Materials and methods

### Insect rearing

The laboratory cultures of *P. puparum* adult wasps were fed with 20% honey solution (v/v, honey/deionized distilled water) and maintained at 25°C, 75% relative humidity and a 14:10 h (light: dark) photoperiod. The wasp host, *Pieris rapae*, was reared on fresh cabbage leaves under the same conditions. Newly pupated *P. rapae* were used as hosts for the wasps to maintain wasp generation in the laboratory (Wang et al., 2017).

### Histone H1 genes identification

We first searched candidate histone H1 genes in the eleven hymenopteran species (Supplementary Table S2) with high-quality genomes using Blastp (E-value ≤ 1e-5), then these sequences were predicted by searching the sequences against the HMM profiles of the Pfam database (Mistry et al., 2021) and SMART (Letunic et al., 2021) (Simple Modular Architecture Research Tool; http://smart.embl-heidelberg.de/) (E-value ≤ 1e-5), using the default parameters. We also checked the complete open reading frame for each candidate. The H1 sequences for Blast search were downloaded from the database HistoneDB 2.0 (Draizen et al., 2016). Finally, each candidate H1 gene was manually inspected and used for analyses.

### Evolutionary analyses of histone H1 genes

The protein sequences of histone H1 genes were aligned by MAFFT v 7.487 (Katoh and Standley, 2013) and filtered by trimAl v1.2 (Capella-Gutiérrez et al., 2009) with the default parameters. The best model for phylogenetic analysis was determined by ModelFinder (Kalyaanamoorthy et al., 2017), and the gene tree was inferred in IQ-Tree v2.0 (Minh et al., 2020) with 1,000 ultrafast bootstrap approximation replicates. We used full-length protein sequences of all H1 variants to calculate pairwise identities between representative hymenopteran orthologs. The species divergence times were obtained from previous studies(Peters et al., 2017; Ye et al., 2022).

### RNA extraction and cDNA synthesis

Total RNA was extracted from male yellow pupae of *P. puparum* using RNAiso Plus (Takara Bio, Otsu, Japan) and the RNA concentration of each sample was measured by Nanodrop 2000 spectrophotometer (Thermo Scientific, Wilmington, DE). Single-strand cDNA was synthesized from the total RNA (1 μg per sample) using the TransScript One-Step gDNA Removal and cDNA Synthesis SuperMix Kit (TransGen Biotech, Beijing, China).

### Gene cloning

The synthesized cDNA was used as a template for polymerase chain reaction (PCR). PCR primers were designed using Primer3Plus (https://www.primer3plus.com) and specific amplification of coding sequences was performed using the KOD One™ PCR Master Mix (Toyobo, Osaka, Japan). The 5′-and 3′-flanking regions of the H1 variant genes were confirmed by 5′ and 3′ rapid amplification of cDNA ends (RACE) using the SMART RACE cDNA amplification kit (Clontech, California, USA). All amplified PCR products were cloned into the pCE2 TA/Blunt-Zero vector (Vazyme, Nanjing, China) and positive cloning was verified by DNA sequencing. Information about the primers used in this study is provided (Supplementary Table S3).

### Gene transcript level analysis

To detect the developmental expression patterns at different stages of the histone H1 variant genes in *P. puparum*, larvae, pupae, and adult wasps were collected and transferred into RNAiso Plus (Takara Bio). Thirty early larvae or five late larvae/pupae/adult wasps were pooled as one biological replicate and each life stage was performed in triplicate. The total RNA extraction and the single-strand cDNA synthesis of different samples were conducted as described above. Quantitative real-time polymerase chain reaction (qRT-PCR) was performed to quantify the expression level of H1 variant genes using specific primers. An absolute standard curve was constructed from a plasmid clone of each gene using specific primers. PCR products were cloned into pCE2 TA/Blunt-Zero vectors (Vazyme) and then sequenced. Standard curves were generated by determination of copy numbers (10^3^–10^8^ copies) of standard plasmid. qRT-PCR was performed using the Bio-Rad CFX 96 Real-Time Detection System (Bio-Rad, Hercules, CA, United States) with SYBR Premix Ex Taq II (Tli RNaseH Plus) (Takara Bio). Thermal cycling conditions were 94°C for 30 s, 40 cycles of 95°C for 5 s, and 60°C for 30 s. Three biological replicates for each group were performed. The equation of y = −3.5095x + 39.678 (y = Ct value; x = the logarithm of plasmid copy number to base 10; *R* ^2^ = 0.9965) was used to calculate the copy number of *PpH1V1*. The equation of y = −3.6725x + 40.492 (y = Ct value; x = the logarithm of plasmid copy number to base 10; *R* ^2^ = 0.9986) was used to calculate the copy number of *PpH1V2*.

The sex and development stage with the highest copy number of both genes was selected to detect the expression in different tissues. Late-stage yellow pupal males were used for dissections. Heads, thoraxes, testes, male accessory glands or the leftover remnants were dissected using a dissecting microscope (Leica, Wetzlar, Germany) then pooled as one biological replicate and performed in triplicate. The extraction of total RNA, the synthesis of single-strand cDNA, and qRT-PCR were conducted for each sample as described above.

### Preparation of dsRNA and RNAi assays

To investigate the functional significance of the H1 variants on *P. puparum*, RNAi knockdown of the genes were utilized to reduce the abundance of the H1 variants, and then the effect of these knockdown on male fecundity were evaluated. RNAi effects on the gene expressions were evaluated at the transcript/mRNA levels.

We used two different, non-overlapping, double-stranded RNA (dsRNA) fragments to exclude off-target effects. The H1 variant genes specific primers and primers targeting enhanced green fluorescent protein gene (*EGFP*; negative control) were designed with added T7 promoter adaptors. All amplified PCR products (150–500 bp) were cloned into pCE2 TA/Blunt-Zero vector (Vazyme) and sequenced. The correct PCR products were used as templates for dsRNA synthesis with the T7 High Yield RNA Transcription Kit (Vazyme), according to the manufacturer’s instructions. Synthesized dsRNA was purified by phenol/chloroform extraction and isopropanol precipitation, dissolved in diethylpyrocarbonate-treated water, and quantified using a NanoDrop 2000 Spectrophotometer (Thermo Scientific) at 260 nm.

46.4 nL of dsRNA (4 × 10^3^ ng/μL) was injected into each larval male using Drummond Nanoject II™ Auto-Nanoliter Injector (Drummond Scientific Company, Broomall, United States). The expression level of the genes in the late-stage yellow pupae developed from injected larvae were quantified by qRT-PCR. 15 uninjected female wasps that had mated with injected male wasps for 48 h were selected to investigate the offspring number and sex ratio.

### Effect of H1 variants on male fertility of *P. puparum*

Major biological parameters including the pupation proportion, adult emergence proportion, offspring number and sex ratio of *P. puparum* were compared among the male RNAi wasps and calculated as described previously (Wang et al., 2017). Reproductive performance of mated females was evaluated in pairings of RNAi or control males with virgin females.

### Polyclonal antibody preparation

We designed the primers (Supplementary Table S3) by the coding sequence of *PpH1V1* or *PpH1V2* with 18 bp extension homologous to the vector ends and amplified the two genes by PCR using the pCE2 TA/Blunt-Zero vector with the full-length cDNA sequences inserted as the templates. Linear pET-32a(+) vectors were generated by FastDigest BamHI and HindIII (Thermo Scientific) restrictions. Then the pET-32a(+)-*PpH1V1* and pET-32a(+)-*PpH1V2* plasmids were constructed by seamless cloning using MonClone™ Hi-Fusion Cloning Mix V2 (Monad Biotech, Suzhou, China). The recombinant plasmids were transformed into *Escherichia coli* bacterial strain BL21(DE3) (TransGen Biotech). A single positive clone containing the inserts of *PpH1V1* or *PpH1V2* was selected and incubated in Luria-Bertani (LB) liquid medium (supplemented with 100 μg/mL ampicillin) in a MaxQ 4000 rotating incubator (Thermo Scientific) at 37°C with 200 rpm rotation until the OD600 of the culture reached 0.6. Isopropyl β-D-1-thiogalactopyranoside (Sangon Biotech, Shanghai, China) was added at 1 mmol/L to induce protein expression, then the bacterial culture was incubated at 16°C with 120 rpm rotation for 16 h. Bacterial cells were collected by centrifugation and disrupted with BugBuster Master Mix (Novagen, San Diego, CA, United States) using standard procedures. The insoluble recombinant His-tagged PpH1V1 protein (43.47 kDa) and PpH1V2 protein (35.24 kDa) were purified using High Affinity Ni-NTA Resin (GenScript, Nanjing, China). To confirm the identity of the recombinant proteins, proteins were separated by SDS-PAGE, transferred to polyvinylidene difluoride membranes (PVDF, Millipore Corporation, Billerica, MA, United States) by an eBlot L1 fast wet transfer system (GenScript), and detected with an anti-His monoclonal antibody (GenScript). Signals were visualized with an enhanced chemiluminescence detection system (Super Signal West Pico Chemiluminescent Substrate; Pierce, Rockford, IL, United States). The recombinant plasmids were submitted to Wuhan Daian Biotechnology Company (Wuhan, Hubei Province, China) for production of purified proteins, which were then used as antigens for immunization of Japanese white rabbits. The obtained polyclonal antibodies to PpH1V1 and PpH1V2 protein were purified from antiserum by the company.

### Immunofluorescence

For immunofluorescence (IF), testes and seminal vesicles from *P. puparum* late-stage yellow pupae, yellow-black pupae, black pupae or adult wasps were dissected, washed, and handled, as described previously (Wu and Luo, 2006). The primary antibodies were rabbit anti-PpH1V1 and rabbit anti-PpH1V2, diluted 1:200 in 0.1 M, pH 7.0 phosphate-buffered saline (PBS; Wisent Biotech, Nanjing, Jiangsu Province, China), containing 0.3% Triton X-100 (Solarbio, Beijing, China) and 5% goat serum (Sangon Biotech). The secondary antibody was Alexa Fluor™ 488 conjugated Goat anti-Rabbit IgG (H + L) Highly Cross-Adsorbed (Invitrogen, Carlsbad, CA, United States), diluted 1:200 in 0.1 M, pH 7.0 PBS (Wisent Biotech). Antibodies for marking sperm flagellum were acetylated α Tubulin Alexa Fluor^®^ 647 antibody (Santa Cruz Biotechnology, Dallas, Texas, United States) and acetylated α Tubulin Alexa Fluor^®^ 488 antibody (Santa Cruz Biotechnology), diluted 1:200 in 0.1 M, pH 7.0 PBS (Wisent Biotech), containing 0.3% Triton X-100 (Solarbio) and 5% goat serum (Sangon Biotech). Cell nuclei were stained with 1 μg/mL 4′,6-diamidino-2-phenylindole (YEASEN Biotech, Shanghai, China). Tissues were analyzed mounted onto slides using SlowFade Gold Antifade Mountant (Invitrogen) and images recorded with a Zeiss LSM 880 confocal microscope (Carl Zeiss SAS, Jena, Germany) and a Zeiss LSM 800 confocal microscope (Carl Zeiss SAS). A stack of consecutive confocal optical sections (Z-stacks) was recorded at 8-bit resolution. Images were merged and scale bars were added using LSM ZEN 3.2 software (Carl Zeiss SAS).

### Transmission electron microscopy (TEM)

Testes of *P. puparum* were dissected from late-stage yellow pupal males using a dissecting microscope (Leica). Seminal vesicles were dissected from 2-day-old adult males. The dissected samples were pre-fixed overnight at 4°C with 2.5% glutaraldehyde (Sinopharm Chemical Reagent Co., Ltd, Shanghai, China) in PBS (0.1 M, pH 7.0). After three washes in PBS for 15 min each, the samples were embedded in 2% agarose diluted in PBS and then fixed with 1% osmium tetroxide (OsO4; SPI, West Chester, PA, United States) in PBS for 1–2 h and washed again for three times. The samples were first dehydrated by a graded series of ethanol (30%, 50%, 70%, 80% for 15 min each), then dehydrated by a graded series of acetone (90%, 95% for 15 min each). In the end, the samples were dehydrated twice by absolute acetone for 20 min each. Samples were infiltrated with a 1:1 mixture of absolute acetone and the final Spurr’s resin (SPI Supplies, West Chester, PA, United States) mixture for 1 h at room temperature, transferred to a 1:3 mixture of absolute acetone and the final resin mixture for 3 h, and then placed into a final Spurr’s resin mixture overnight. Finally, each specimen was placed in an Eppendorf tube containing Spurr’s resin, incubated at 70°C for more than 9 h, and then sectioned using an EM UC7 ultramicrotome (Leica). Ultrathin sections were double-stained with uranyl acetate (SPI Supplies) for 5 min and alkaline lead citrate (Electron Microscopy Sciences) for 10 min and then were observed using an H-7650 TEM (Hitachi, Tokyo, Japan) at an accelerating voltage of 80 kV.

### Sperm viability assay

The sperm viability was determined using the Live/Dead sperm viability kit (Invitrogen) as described previously (Zhang et al., 2018). Two fluorescence probes that bind to DNA were used. The first probe (SYBR-14) emits green fluorescence and is actively incorporated by living cells, and the second probe (propidium iodide, PI) emits red fluorescence and can only enter cells with damaged membranes. Seminal vesicles from individual males at 2–3 days post emergence were dissected, washed three times and sperm were collected in 5 µL HEPES solution (10 mM HEPES, 150 mM NaCl, pH 7.4). First, 5 µL SYBR-14 working solution (2 µL SYBR-14 stock in 98 µL HEPES solution) was added to the semen solution, followed by incubation for 10 min in the dark. Then, the solution was incubated with 2 µL PI for another 7 min. Images were recorded with a Zeiss LSM 800 confocal microscope (Carl Zeiss SAS). Sperm viability was obtained for each sample by calculating the percentage of live sperm in the total number of sperm counted. Number of seminal vesicle replications for examining sperm viability is three. A stack of consecutive confocal optical sections (Z-stacks) was recorded at 8-bit resolution. Images were merged, and scale bars were added using LSM ZEN 3.2 software (Carl Zeiss SAS).

## Data Availability

The datasets presented in this study can be found in online repositories. The names of the repository/repositories and accession number(s) can be found in the article/Supplementary Material.
