# Supplementary material for: A rapidly evolving single copy histone H1 variant is associated with male fertility in a parasitoid wasp
Source: Front Cell Dev Biol. 2023 May 31;11:1166517. doi: 10.3389/fcell.2023.1166517 (PMC10264595; doi:10.3389/fcell.2023.1166517)
Supplement: Supplementary file 1 [file DataSheet1.pdf]

Supplementary Material

A rapidly evolving single copy histone H1 variant is associated with male fertility in a parasitoid wasp

Bo Yuan, Yi Yang, Zhichao Yan, Chun He, Yu H. Sun, Fei Wang, Beibei Wang, Jiamin Shi, Shan Xiao, Fang Wang, Qi Fang, Fei Li, Xinhai Ye*, Gongyin Ye

*** Correspondence:** Xinhai Ye, yexinhai@zju.edu.cn

Supplementary Figure S1. PCR amplication of *PpH1V1* and *PpH1V2* full-length cDNA in *P. puparum*.

Supplementary Figure S2. Expression patterns of PpH1V1 and PpH1V2 in germ cells of testes.

Supplementary Figure S3. Cysts with germ cells undergoing division.

Supplementary Figure S4. *PpH1V1* knockdown does not affect gene transcription during early spermatogenesis.

Supplementary Figure S5. *PpH1V1* knockdown results in irregular shape of sperm nuclei.

Supplementary Figure S6. *PpH1V1* knockdown does not affect the density of sperm in seminal vesicles.

Supplementary Table S1. Sequence information of the full-length cDNA of H1 variants.

Supplementary Table S2. The information of eleven hymenopteran species.

Supplementary Table S3. Primers used in this study.

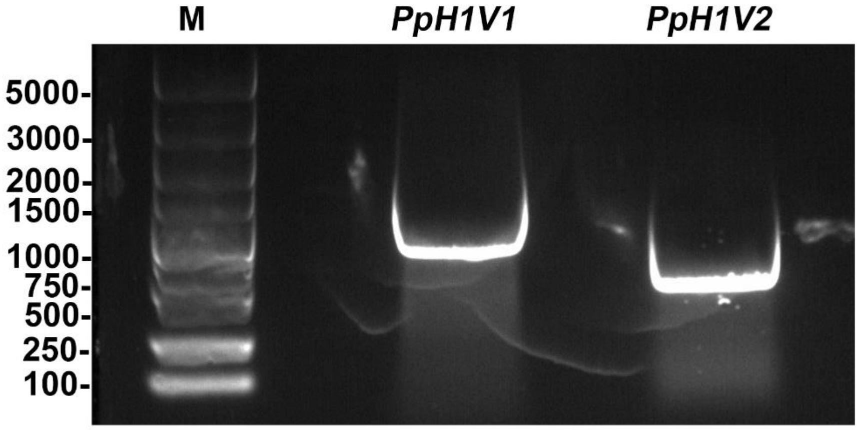

Supplementary Figure S1 PCR amplication of *PpH1V1* and *PpH1V2* full-length cDNA in *P. puparum*. The amplified bands are corresponding to the pairs of primers listed in Supplementary Table S3.

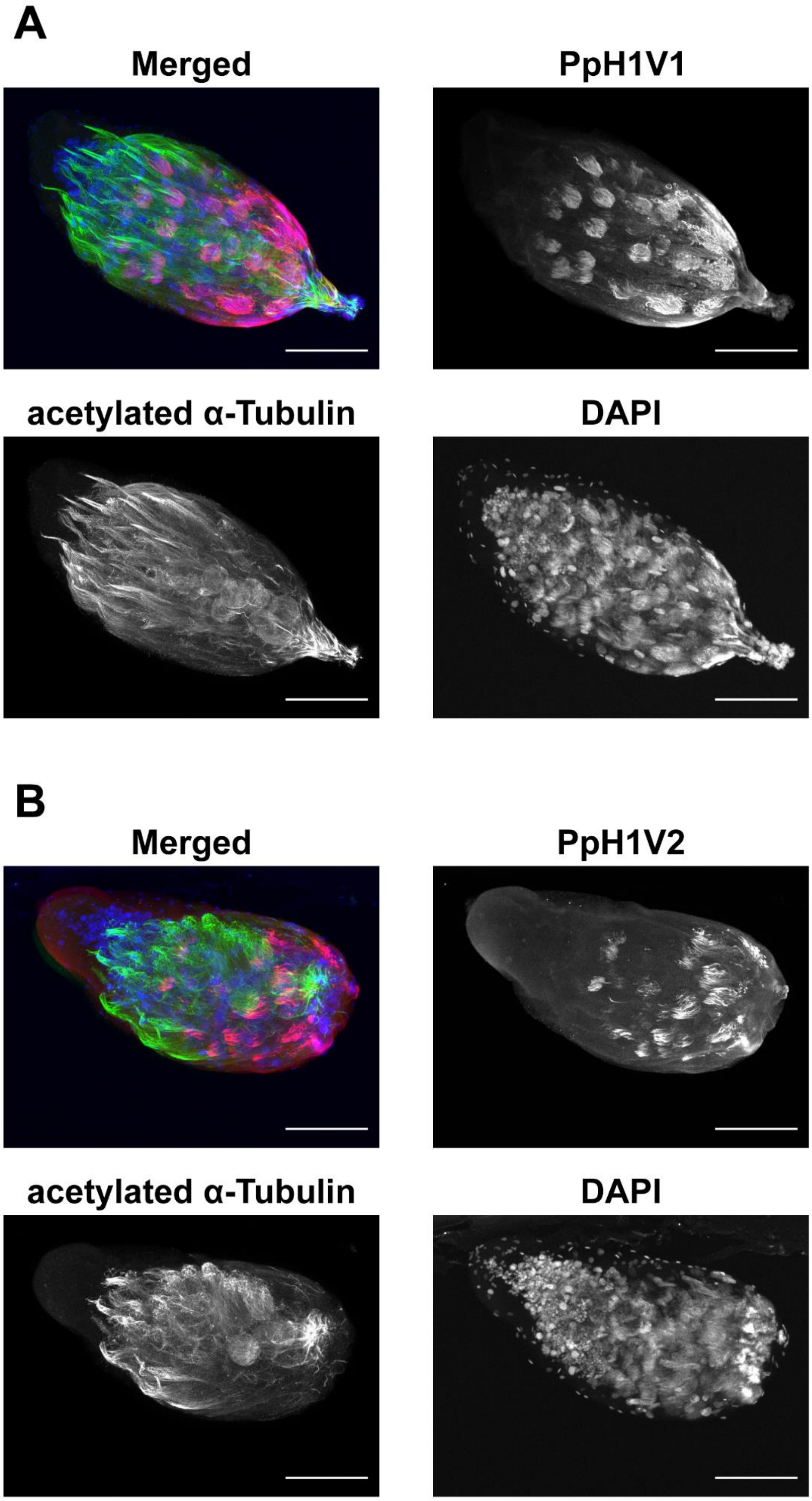
A
Merged
PpH1V1
acetylated α-Tubulin
DAPI
B
Merged
PpH1V2
acetylated α-Tubulin
DAPI

Supplementary Figure S2 Expression patterns of PpH1V1 and PpH1V2 in germ cells of testes. Immunofluorescence staining showing the testes of yellow-black pupal males. PpH1V1 **(A)** or PpH1V2 **(B)** was detected using a rabbit anti-PpH1V1 or a rabbit anti-PpH1V2 polyclonal antibody and goat anti-rabbit IgG (H+L) with Alexa Fluor™ 488 secondary antibody (shown in red). Flagellum was stained with acetylated α Tubulin Alexa Fluor® 647 antibody (shown in green). Nuclei was stained with DAPI (shown in blue). Images are shown in greyscale for single channels. Five biological replicates were performed for each measurement. Scale bars correspond to 100 μm.

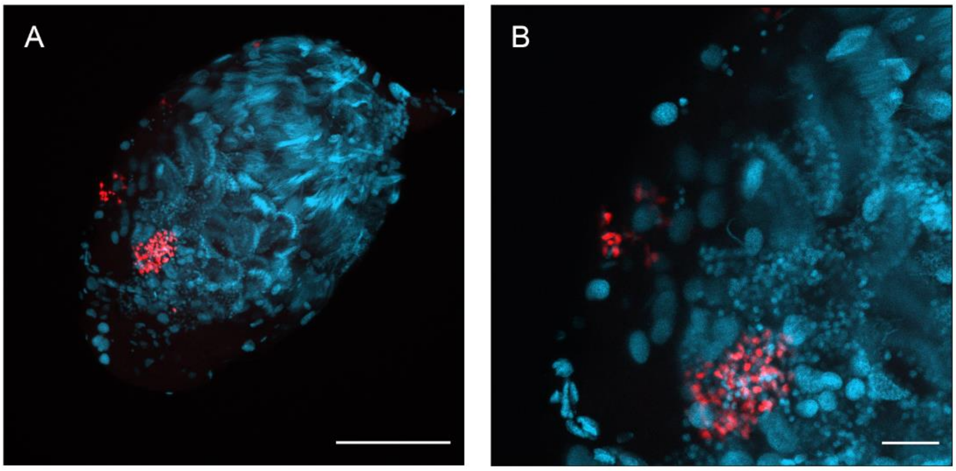

Supplementary Figure S3 Cysts with germ cells undergoing division. **(A)** A testis from a late-stage yellow pupal testis containing cysts with germ cells undergoing division, as indicated by the presence of H3S10p stained with Phospho-Histone H3-S10 Rabbit antibody (showed in red). Nuclei was stained with DAPI (showed in light blue). **(B)** The local enlarged image of **(A)**. Three biological replicates were done. Scale bars correspond to 100 μm in **(A)** and 20 μm in **(B)**.

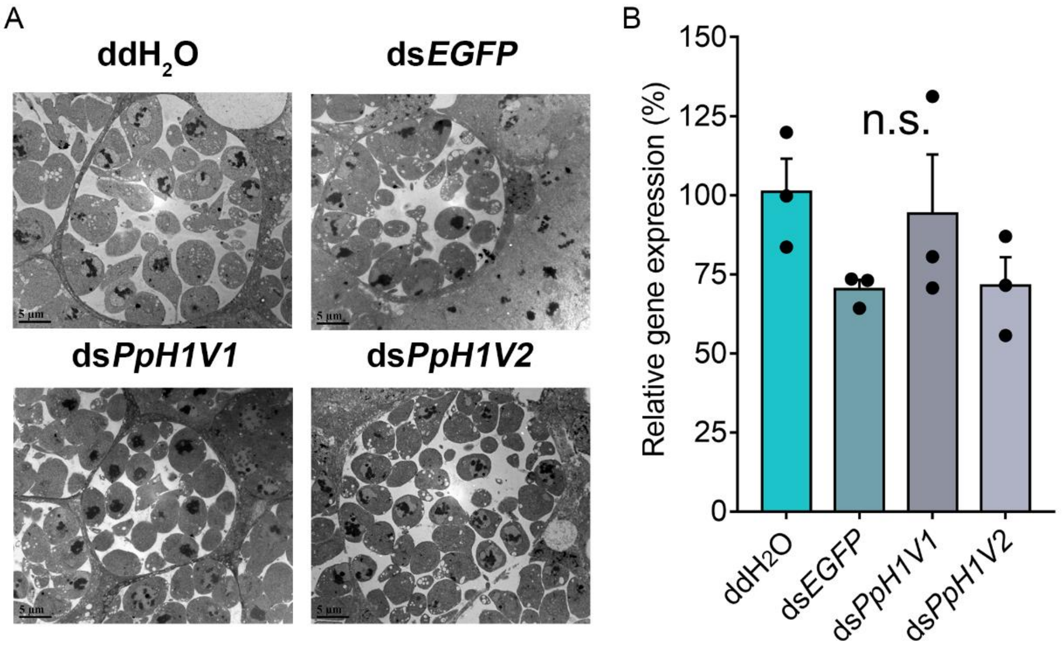

Supplementary Figure S4 *PpH1V1* knockdown does not affect gene transcription during early spermatogenesis. **(A)** TEM images of small cyst of germ cells taken from the late-stage yellow pupal testes. For each measurement at least three biological replicates were done. Scale bars correspond to 5 μm. **(B)** Quantitative analysis of the germline-specific marker *Vasa* expressions in male pupae. For each measurement three biological replicates were done. The qRT-PCR data were presented as mean ± standard error. Differences between groups were analyzed by one-way ANOVA with Tukey's multiple comparisons test; n.s.: not significantly different.

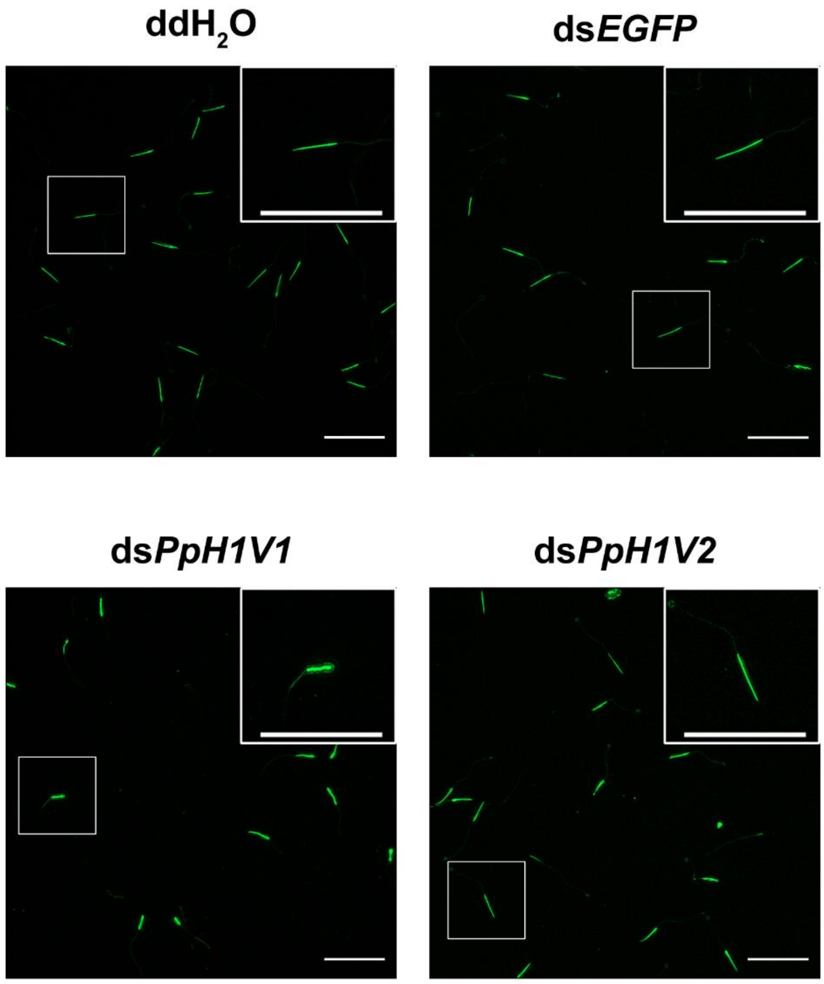

Supplementary Figure S5 *PpH1V1* knockdown results in irregular shape of sperm nuclei. Sperm nucleus were shown in green. For each measurement at least three biological replicates were done. Scale bars correspond to 100 µm.

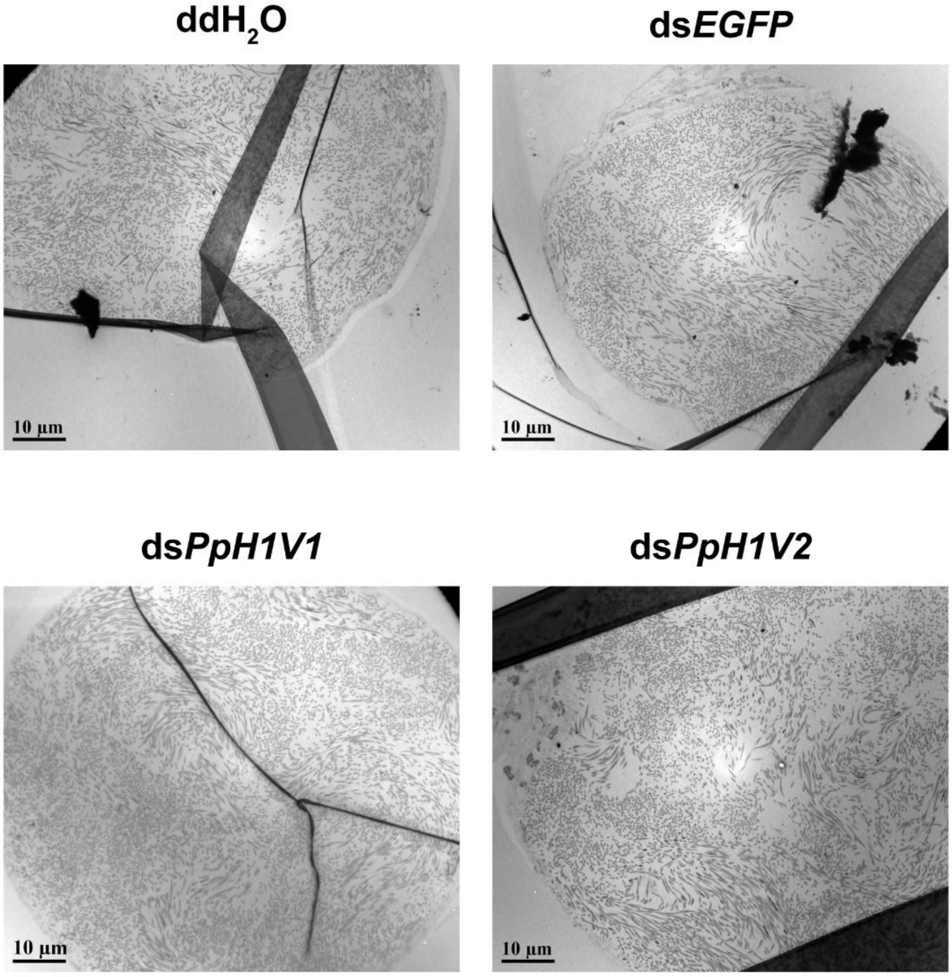

Supplementary Figure S6 *PpH1V1* knockdown does not affect the density of sperm in seminal vesicles. TEM images of sections of seminal vesicles taken from RNAi-treated 2-day-old adult males. For each measurement at least three biological replicates were done. Scale bars correspond to 10 µm.

Supplementary Table S1 Sequence information of the full-length cDNA of H1 variants.

Name	Sequence information
PpH1V1	GAGTCAACCAAGTTCCGTCGACATAACCG<br>GACCTGTTCTCCCTCGTCTCCCCTTCACC<br>AGGACTCCTTCCTTCTCGGCCGACATTTA<br>ACGGAAAAATTCTCGGATCGACCGCGCA<br>GCATGGAAAATTATCAGTGACCTGGAAGA<br>AAAAAAAACGGAAATTTGGAAAAGCCGC<br>TGCGGAGAAAAGGAAGCCAAGGAACTTC<br>GCGAGCGACGAGAGCAGCCCCTCAGGAT<br>GAAGACGAAGAAGTCGCAGCCGAAGATG<br>TCCGGACTGGTCGTCGCTGCCCTCAGGAA<br>CCTGAGGGACGCCCATGGCTCCACCGCC<br>AAGGAGATCATGAAGTACATCATGGCCGA<br>GTACAACGCGTCCGAGGCTACCGTTCAAC<br>GTCAGCTGAGGACAGCTTTGAAGCGCGG<br>TGTTGAATACGGGATCTTGAAAAAGACGA<br>CCGCCGGCTACAGCCTGAACACGGATGCC<br>GAAGTCATCGGCCCGCTCGACTTGGCGCA<br>GATGGACAGCTGCGGCAAGCGACGAAAA<br>CGTCACGGCTGCAGCATGCGTCGCAAGC<br>GCTCGGCCTGCGGCATGAAGAAGAAGCG<br>CAAGCGCGCGTCTTGTGCTCCAAGGCGC<br>CGGCGCAAGAAGAACCCGTGCGGCATGT<br>GCGCAGCTCGCGGTCGCAAGGGTGACTG<br>CAGCGCGACCCCTGTAGAGCTGGTGCGC<br>GAAGGTACGCCCAACCCTGAAGAGGAGG<br>CAGCCTCGAACGAGGCCAATCGCGAGAC<br>CGGCGAGAGACGTTCGCGCAGTCGCAGC<br>ACCAGCCGGGCTAAGCAGACACGGAATC<br>GATCCAGGAGCCAAAGCATGGCCAAGAG<br>CCAGTACTCCGGCAGCGAGAACGAGGAA<br>GATAGGCAGGAGGACGACATGATGTAGA<br>GCGATTGGCTGATTCGACGTTTTGTGATC<br>CGTGTTGTTCTCATTTTCGATTCTAGATTT<br>TTAGTGACAGTTTGACAAACATGATGTGT<br>CGTTGTTGGATATATTTTTCTATTTTGGACT<br>CGTGTAAATTTTTTCGATTATATATTTAGAG<br>GATGAATAAATTCCCTGTGGCTTTCGTGC<br>GTAAAAAAAAAAAAAAAAAAAAAAAAAA<br>AAAAAAAAA
PpH1V2	GAGTTGCGGCTCGGGCTTTCAAACAAAAT

	TCTCGTGACTCCCAGCTCTCTCGTCATCC<br>CGCGTATTTTTACAGTTCCCTCTAGAAAA<br>AACCTTCTCGGAGAAAAAATGCCAGCCG<br>CAGCCGAGGGAACGACGAACCGGACGA<br>ACAGCGGAGATTCGTCAAGGCTCAAAAT<br>CGCGGCGAAGGCTGTTGGTCAGGTTATGG<br>CTGCTCTGAAGAACTCGAAGAACACGAC<br>TGGACCGACTATGACGGAGATCGTTAAAT<br>TCATCTCCGGCGCGCTAATCAAGCCTGCG<br>ACGAAACGCCAGGTCGTCACGGCTCTGA<br>AACGTGGCGTCGAGTTCGGTATCCTCAAG<br>CGCAAAAAGGGCCACTACCTGATCTCTTC<br>GCCCGAGGAGAACTTCTCGAAGCTGCCA<br>CTACCCAAGAAAACTACGAAGACTCGAG<br>GAAAGCTTCTGGAGAAGGTGTCGAAACG<br>AGTTCGTGCTCCGCTGAAGTCGAGGCGTC<br>CTAGAGCTGGAGGCAGAGATCTCATGGAC<br>GACTTTCCATCACCACCTTTTCCACCAAA<br>GTTCTGAAGATTCGAAGGTGAAGCTTGTG<br>TGAAACGTGAAGAATTTTTAAATCGTACA<br>CAGTGATTCGACGAAATAACAGTATACCT<br>TGTGAACTTGCGCTAAATTTCGGTTGTGA<br>CAATACAAACAAAAAAGCTATACGATCGA<br>AAAAAAAAAAA

Supplementary Table S2 The information of eleven hymenopteran species.

Family	Species	Data Source
Ptermoalidae	*Pteromalus puparum*	NCBI (GCA_012977825.2)
	Nasonia vitripennis	NCBI (GCA_009193385.2)
	Anisopteromalus calandrae	InsectBase 2.0
	Pachycrepoideus vindemmiae	InsectBase 2.0
Agaonidae	*Ceratosolen solmsi*	NCBI (GCA_000503995.1)
Encyrtidae	*Copidosoma floridanum*	NCBI (GCA_000648655.2)
Trichogrammatidae	*Trichogramma pretiosum*	NCBI (GCA_000599845.3)
Braconidae	*Microplitis demolitor*	NCBI (GCA_000572035.2)
Formicidae	*Solenopsis invicta*	NCBI (GCA_016802725.1)
Apidae	*Apis mellifera*	NCBI (GCA_003254395.2)
Tenthredinidae	*Athalia rosae*	NCBI (GCA_000344095.2)

Supplementary Table S3 Primers used in this study.

Primer usage	Primer name	Primer sequence (5'- 3')
Cloning	PpH1V1-5’RACE-GSP1	CGTTGTACTCGGCCATGATGTACTTCA
	PpH1V1-5’RACE-GSP2	GCGGTCGTCTTTTTCAAGATCCCGTAT
	PpH1V1-5’RACE-GSP3	CTTGCGCTTCTTCTTCATGCCGCAG
	PpH1V1-3’RACE-GSP1	ATACGGGATCTTGAAAAAGACGACCGC
	PpH1V1-3’RACE-GSP2	CTGCGGCATGAAGAAGAAGCGCAAG
	PpH1V1-3’RACE-GSP3	AAGATAGGCAGGAGGACGACATGATGT
	PpH1V2-5’RACE-GSP1	GTCGTGTTCTTCGAGTTCTTCAGAGCA
	PpH1V2-5’RACE-GSP2	AAGAGATCAGGTAGTGGCCCTTTTTGC
	PpH1V2-5’RACE-GSP3	TTGGTGGAAAAGGTGGTGATGGAAAGT
	PpH1V2-3’RACE-GSP1	GTTAAATTCATCTCCGGCGCGCTAATC
	PpH1V2-3’RACE-GSP2	CGAAGCTGCCACTACCCAAGAAAACTA
	PpH1V2-3’RACE-GSP3	ACTTTCCATCACCACCTTTTCCACCAA
	PpH1V1-cDNA-F	GAGTCAACCAAGTTCCGTCGACA
	PpH1V1-cDNA-R	ACGCACGAAAGCCACAGGGA
	PpH1V2-cDNA-F	GAGTTGCGGCTCGGGCTTTC
	PpH1V2-cDNA-R	CGATCGTATAGCTTTTTTGTTTGTATTG
dsRNA	dsPpH1V1-1-F	taatacgactcactatagggAAGAAGTCGCAGCCGAAGAT
	dsPpH1V1-1-R	taatacgactcactatagggCATCCGTGTTCAGGCTGTAG
	dsPpH1V1-2-F	taatacgactcactatagggCTGCGGCATGAAGAAGAAG
	dsPpH1V1-2-R	taatacgactcactatagggGATTCCGTGTCTGCTTAGCC
	dsPpH1V2-1-F	taatacgactcactatagggTCGTCAAGGCTCAAAATCGC
	dsPpH1V2-1-R	taatacgactcactatagggCGCGCCGGAGATGAATTTAA
	dsPpH1V2-2-F	taatacgactcactatagggAATCAAGCCTGCGACGAAAC
	dsPpH1V2-2-R	taatacgactcactatagggACTCGTTTCGACACCTTCTCC
	dsEGFP-F	taatacgactcactatagggCAGCAGGACCATGTGATCGCGC
	dsEGFP-R	taatacgactcactatagggAAGGGCGAGGAGCTGTTCACCG
qRT-PCR	qPpH1V1-F	GCGGTGTTGAATACGGGATC
	qPpH1V1-R	TTGCGCTTCTTCTTCATGCC
	qPpH1V2-F	CGGTATCCTCAAGCGCAAAA
	qPpH1V2-R	TCGTCCATGAGATCTCTGCC
	qPp18S-F	CGAGCGATGAACCGACAG
	qPp18S-R	CGGGGAGGTAGTGACGAA
	qPpVasa-F	CCAACTCCCGTGCAGAAGTA
	qPpVasa-R	TCTCGATCTTTCGCCAGCAA
Prokaryotic expression	PpH1V1-pET32a(+)-BamHI	atggctgatatcggatccATGAAGACGAAGAAGTCGCAGC
	PpH1V1-pET32a(+)-HindIII	gagtgcggccgcaagcttCATCATGTCGTCCTCCTGCCTA
	PpH1V2-pET32a(+)-BamHI	atggctgatatcggatccATGCCAGCCGCAGCCGAGGGAA
	PpH1V2-pET32a(+)-HindIII	gagtgcggccgcaagcttGAACTTTGGTGGAAAAGGTGGT
